# Diabetes and Tuberculosis: harmonization of population-based datasets

**DOI:** 10.21203/rs.3.rs-11311043/v1

**Published:** 2026-10-14

**Authors:** Adriana Gutierrez Yllu, Bindu Edupulapati, Rodrigo M Carrillo-Larco

## Abstract

BACKGROUND Leveraging existing datasets is an efficient and effective mean of advancing health research. However, finding and pooling these datasets for certain comorbid conditions, such as diabetes and tuberculosis, can be challenging because most open-access datasets may focus on only one of these conditions. AIMS To develop a data infrastructure that can be utilized to study diabetes and tuberculosis in diverse populations. METHODS We cleaned, harmonized, and pooled three population-based datasets and one with electronic medical records: i) Hispanic Community Health Study/Study of Latinos (HCHS/SOL); ii) Coronary Artery Risk Development in Young Adults (CARDIA); iii) National Health and Nutrition Examination Survey (NHANES); and iv) All of Us (AoU) Research Program. Diabetes was defined as a combination of self-reported diagnosis, blood tests, and ICD-10 codes; tuberculosis was defined using self-reported diagnosis and ICD-10 codes. We provided user guides and analytical code for researchers to develop analytical datasets to study diabetes and tuberculosis. RESULTS There were 116 unique individuals who met criteria for both diabetes and tuberculosis: 20-year-round CARDIA (n = 19); baseline evaluation of SOL (n = 89); and two NHANES waves (n = 8). For AoU, we identified 253 unique individuals who had a diabetes diagnosis prior to the tuberculosis diagnosis, 18 who had both diagnoses recorded simultaneously, and 213 whose tuberculosis diagnosis was registered before the diabetes diagnosis. CONCLUSIONS Leveraging available data resources, which are predominantly utilized for research on noncommunicable diseases, is feasible to expedite the field and integrate both noncommunicable and infectious diseases.

### INTRODUCTION

Although the association between diabetes and tuberculosis has been well-established,[1] as have the epidemiological and social contexts that favor both diseases together with their health and economic consequences,[2,3] their concurrent study has often relied on new data collection or leveraging electronic medical records (EMR). This contrasts with studies about diabetes and other cardiometabolic conditions, which frequently leverage open-source and population-based, large datasets such as cohorts (e.g., Framingham) and surveys (e.g., NHANES). Potential reasons for this include the apparent lack of tuberculosis-related information in cardiometabolic datasets, and the complexities associated with navigating the intricacies of collating, cleaning, and harmonizing these cardiometabolic datasets. Therefore, to provide researchers with tools they can use to leverage strong, well-established cardiometabolic datasets to study diabetes and tuberculosis, we cleaned and harmonized three prime cardiometabolic datasets that have been published in thousands of papers across the spectrum of chronic, noncommunicable diseases. This will make it easier to incorporate tuberculosis outcomes. Additionally, we provide guidelines on how to leverage a new large data resource with access to EMR to study diabetes and tuberculosis (All of Us (AoU) research program). Our goal is to enhance access to data resources that are typically reserved for research on noncommunicable diseases, providing data at the intersection of diabetes and tuberculosis.

### METHODS

## Data Sources

We utilized three open-access datasets primarily employed in the study of cardiometabolic conditions: i) Hispanic Community Health Study/Study of Latinos (HCHS/SOL);[4,5] ii) Coronary Artery Risk Development in Young Adults (CARDIA);[6,7] and iii) National Health and Nutrition Examination Survey (NHANES).[8] SOL and CARDIA are open-access and accessible through BioLINCC,[9] the National Heart, Lung, and Blood Institute (NHLBI) data repository. Access to these datasets needs the submission of an analysis plan and IRB approval (e.g., exception due to non-human subjects research). NHANES is freely available to anyone, and all datasets can be downloaded from its website without any restrictions.[8]

We also used the AoU research program.[10,11] This data resource, supported by the National Institutes of Health (NIH), comprised a comprehensive health questionnaire and EMR from volunteers in the United States (US). Access to AoU requires registration, account creation, and completion of several mandatory courses to familiarize oneself with AoU and responsible research ethics. AoU data can only be accessed through their workbench. AoU cannot be merged with other data resources outside the AoU workbench. Despite this limitation, which prevents its integration with the population-based datasets, we still selected AoU because it can serve as a parallel dataset to replicate or validate findings from the population-based datasets.

In all cases, we utilized the datasets available at their respective data repositories up to December 2025. We did not have privileged access. Researchers can utilize our methods, guides, and code, to work with the datasets available from BioLINCC and NHANES, as well as from AoU. Due to the data sharing agreements, we are unable to share the datasets. However, our code and guides are sufficient for researchers to seamlessly generate analytical datasets for studying both diabetes and tuberculosis.

### Research Population

Detailed information regarding the study populations and sampling strategies used by SOL,[4,5] CARDIA,[6,7] NHANES,[8] and AoU[10,11] is available elsewhere.

SOL is a community-based prospective cohort of Hispanic/Latino adults (18–74 years at baseline) conducted at four US centers: the Bronx, New York; Chicago, Illinois; Miami, Florida; and San Diego, California. Hispanic/Latino ancestry was determined through self-reports. SOL used a two-stage probability sample of households, with oversampling of people aged ≥ 45 years, a common practice to assess cardiovascular risk factors and chronic diseases that are more prevalent in middle-aged adults. The SOL baseline evaluation took place in 2008–2011, while the second visit occurred in 2014–2017. Only data from these two visits were available at BioLINCC and herein utilized.

CARDIA is a community-based prospective cohort that used a random sampling approach from census tracts in four urban sites: Birmingham, Alabama; Chicago, Illinois; Minneapolis, Minnesota; and Oakland, California. The sample was designed to achieve balanced subgroups of race, gender, education, and age groups. At baseline, targeted participants were aged 18–30 and recruited between 1985–1986. Due to the availability of tuberculosis-related data, we utilized the 10-year (1995–1996), 15-year (2000–2001), and 20-year (2005–2006) rounds.

NHANES is a population-based survey with a complex, multistage, probability sampling design aimed at producing nationally representative information of the civilian, non-institutionalized US population. Due to the availability of tuberculosis-related data, we utilized the rounds conducted in 1999–2000 and 2011–2012.

AoU was founded in 2018 with the objective of recruiting people from demographic groups usually underrepresented in biomedical research. Thus, people from underrepresented populations who choose to enroll have been prioritized for clinical evaluations and the collection of biospecimens. The sample is non-probabilistic, and any adult (≥ 18 years) can join AoU virtually. Participants are asked to provide access to their EMR.

In summary, these four data resources were utilized due to their inclusion of variables pertaining to both diabetes and tuberculosis. We verified the questionnaires, protocols, and documentation of other NHLBI cohorts available at BioLINCC (e.g., MESA and ARIC) but were unable to locate information on both conditions. We refrained from searching data from trial studies or studies that exclusively relied on hospital data. This decision was made because our focus was on population-based samples or the general population. While SOL, CARDIA, and NHANES employed some sort of probability sampling technique, AoU did not. Nonetheless, AoU targets a diverse population recruited from across the US prioritizing underserved populations (i.e., population-based sample albeit not randomly selected).

### Data Handling

## Variables

We inspected the questionnaires of the selected data resources searching for any variable related to diabetes and tuberculosis; these include self-reported diagnosis, having been prescribed medication, or tests such as fasting glucose, HbA1c and for tuberculosis, a skin test. This process was conducted by one researcher (RMC-L) and verified by two others (AG-Y and BE).

### Dataset Management

We followed a systematic approach starting with variable identification, ending with a consolidated dataset including observations from SOL, CARDIA, and NHANES. Concurrently, we replicated an analogous methodology with AoU, albeit this dataset was not integrated with SOL, CARDIA, and NHANES due to restrictions in exporting AoU data. Also, because in comparison to SOL, CARDIA and NHANES, AoU used a different sampling design. Detailed information pertaining to this process is accessible within the user guide accompanying this paper. A summary follows.

Having identified the variables of interest, we then identified the distinct datasets where these variables were stored. SOL, CARDIA, NHANES and AoU offer unique datasets that can be merged with the unique subject identifier. For instance, they may have a dataset containing health background questionnaires and another dataset with blood test results. We cleaned each of these datasets to retain only the variables of interest. Next, we performed cleaning and harmonization of the variables. For instance, we had decided to code men as 1 and women as 2; self-reported diagnosis questions were coded as no (0) and yes (1); and blood glucose tests were set to mg/dl. Additionally, we standardized the names of the variables. Thus, we created individual datasets containing solely the relevant variables and in a homogenous format. This facilitated seamless appending between datasets, because they all share the same names, levels, and units.

## Deliverables

The objective of this work is to condense our experience in identifying, compiling, cleaning, and harmonizing diabetes and tuberculosis data from population-based health data sources to facilitate their utilization in the study of the diabetes-tuberculosis comorbidity. The goal is for researchers to utilize this document to use SOL, CARDIA, NHANES, and AoU to investigate diabetes and tuberculosis. To further facilitate this, we provide the following resources:

- Four RMarkdown files (.Rmd): Three of these files illustrate the cleaning process of SOL, CARDIA, and NHANES, respectively, while the fourth file shows the process of appending these three datasets to create a large data resource that leverages the three datasets.
- Four PDF files: These files present the outputs of the RMarkdown files in a human-readable format. This is an ideal resource for people without programming skills who are not familiar with RMarkdown files.
- Two Word documents: These documents include a user guide with exhaustive details of all the procedures we followed from selecting the datasets to cleaning and harmonizing. The other Word document presents the analytical code to derive the dataset from AoU. Data from the AoU cannot leave the AoU workbench. Nevertheless, to continue providing our readers with practical and usable tools, we are sharing the analytical code in plain text (no data are shown) for users to copy and paste onto their AoU workbench.
- One Excel file: This document displays the variable mapping. It includes the original variable names, as found in the datasets, their meaning, and levels. This file also includes the codebook (Supplementary Table 1) for the pooled dataset (CARDIA, SOL, NHANES).

These resources are made available to the research community to enable them to leverage these data resources to study diabetes and tuberculosis. We undertook the challenge of exploring these data resources to alleviate researchers' concerns about considering using these datasets for diabetes-tuberculosis research. In doing so, we have shown that these resources, primarily used to study chronic noncommunicable diseases (CARDIA, SOL, NHANES), can be further utilized to study a common comorbidity (diabetes-tuberculosis). The released documents are intended to provide researchers with practical tools to streamline their work and initiate immediate diabetes-tuberculosis research.

### Ethics

We analyzed open-access data without including any personal identifiers. We classified this research as non-human subjects research and did not seek IRB clearance.

### RESULTS

## SOL, CARDIA and NHANES

In three rounds of CARDIA, which included data on both diabetes and tuberculosis, we defined diabetes as any of the following criterion: self-reported diagnosis, fasting plasma ≥ 126 mg/dl, HbA1c ≥ 6.5%, or post-prandial glucose tolerance test ≥ 200 mg/dl. In the 10-year-round, four individuals met the diabetes criteria and self-reported tuberculosis diagnosis; in the 15-year-round, there were five cases; and in the 20-year-round there were 19 cases.

Using the same diabetes definition in conjunction with self-reported tuberculosis diagnosis, there were 89 individuals who met these criteria at the baseline evaluation of SOL, while 17 individuals met these criteria at the second follow-up round.

Similarly, using the same diabetes and tuberculosis definitions, there were three cases in the NHANES 1999–2000 survey and five cases in the NHANES 2011–2012 survey.

In total, there were 142 cases of diabetes and tuberculosis across the rounds of SOL, CARDIA, and NHANES. Focusing on unique individuals only, there were 116 who met the criteria for both diabetes and tuberculosis, leveraging data from the 20-year-round of CARDIA (n = 19), the baseline evaluation of SOL (n = 89), and two waves of NHANES (n = 8) (Table 1). Notably, NHANES is a cross-sectional national health survey, which means that only new individuals are recruited at each round. This allows for the pooling of different rounds without duplicate subjects.

Table 1
Description of the analytical sample in the pooled dataset (SOL, CARDIA, NHANES).

| Variables | Total N = 116[1] | CARDIA Y20 N = 19[1] | HCHS/SOL V1 N = 89[1] | NHANES 1999 N = 3[1] | NHANES 2011 N = 5[1] |
|---|---|---|---|---|---|
| Age | 53 (44, 61) | 26 (21, 28) | 54 (49, 61) | 67 (66, 74) | 59 (54, 61) |
| Sex | | | | | |
| *Men* | 44 / 116 (38%) | 4 / 19 (21%) | 35 / 89 (39%) | 3 / 3 (100%) | 2 / 5 (40%) |
| *Women* | 72 / 116 (62%) | 15 / 19 (79%) | 54 / 89 (61%) | 0 / 3 (0%) | 3 / 5 (60%) |
| Race/Ethnicity | | | | | |
| *Mexican/Other Hispanic* | 55 / 116 (47%) | 0 / 19 (0%) | 53 / 89 (60%) | 2 / 3 (67%) | 0 / 5 (0%) |
| *Non-Hispanic Black* | 22 / 116 (19%) | 18 / 19 (95%) | 0 / 89 (0%) | 1 / 3 (33%) | 3 / 5 (60%) |
| *Non-Hispanic White* | 1 / 116 (0.9%) | 1 / 19 (5.3%) | 0 / 89 (0%) | 0 / 3 (0%) | 0 / 5 (0%) |
| *Other/Multiracial* | 2 / 116 (1.7%) | 0 / 19 (0%) | 0 / 89 (0%) | 0 / 3 (0%) | 2 / 5 (40%) |
| *Missing* | 36 / 116 (31%) | 0 / 19 (0%) | 36 / 89 (40%) | 0 / 3 (0%) | 0 / 5 (0%) |
| Education | | | | | |
| *<High School* | 31 / 116 (27%) | 0 / 19 (0%) | 28 / 89 (31%) | 3 / 3 (100%) | 0 / 5 (0%) |
| *High School Grad* | 50 / 116 (43%) | 8 / 19 (42%) | 42 / 89 (47%) | 0 / 3 (0%) | 0 / 5 (0%) |
| *Some College/AA* | 28 / 116 (24%) | 9 / 19 (47%) | 18 / 89 (20%) | 0 / 3 (0%) | 1 / 5 (20%) |
| *College+* | 5 / 116 (4.3%) | 1 / 19 (5.3%) | 0 / 89 (0%) | 0 / 3 (0%) | 4 / 5 (80%) |
| *Refused/DK* | 2 / 116 (1.7%) | 1 / 19 (5.3%) | 1 / 89 (1.1%) | 0 / 3 (0%) | 0 / 5 (0%) |
| Self-reported diabetes | 91 / 116 (78%) | 14 / 19 (74%) | 72 / 89 (81%) | 1 / 3 (33%) | 4 / 5 (80%) |

[1] Median (Q1, Q3); n / N (%). NA: not available.

| Variables | Total<br><br>N = 116[1] | CARDIA Y20<br><br>N = 19[1] | HCHS/SOL V1<br><br>N = 89[1] | NHANES 1999<br><br>N = 3[1] | NHANES 2011<br><br>N = 5[1] |
|---|---|---|---|---|---|
| Age told DM | 44 (35, 49) | 38 (34, 43) | 45 (38, 50) | 43 (43, 43) | NA (NA, NA) |
| Diabetes Mellitus, insulin | 23 / 80 (29%) | 0 / 0 (NA%) | 22 / 72 (31%) | 1 / 3 (33%) | 0 / 5 (0%) |
| Diabetes Mellitus, oral medication | 64 / 96 (67%) | 11 / 13 (85%) | 50 / 78 (64%) | 0 / 1 (0%) | 3 / 4 (75%) |
| Age tuberculosis diagnosis | 27.50 (27.00, 33.50) | 27.50 (27.00, 33.50) | NA (NA, NA) | NA (NA, NA) | NA (NA, NA) |
| Tuberculosis treatment ever | 71 / 97 (73%) | 0 / 0 (NA%) | 66 / 89 (74%) | 2 / 3 (67%) | 3 / 5 (60%) |
| Fasting (minutes) | 790 (45, 829) | 810 (780, 847) | NA (NA, NA) | 9 (7, 50) | 14 (10, 39) |
| Fasting Glucose (mg/dl) | 125 (102, 181) | 113 (105, 173) | 128 (99, 187) | 109 (109, 109) | 128 (107, 144) |
| HbA1c (%) | 7.15 (6.30, 8.35) | NA (NA, NA) | 7.15 (6.30, 8.35) | 7.90 (6.50, 10.10) | 6.40 (6.30, 7.40) |
| Total cholesterol (mg/dL) | 193 (162, 231) | NA (NA, NA) | 193 (162, 231) | NA (NA, NA) | NA (NA, NA) |
| LDL cholesterol (mg/dL) | 108 (86, 138) | 87 (68, 108) | 122 (89, 150) | 187 (187, 187) | 117 (61, 136) |
| Weight (kg) | 80 (70, 93) | 86 (70, 108) | 79 (71, 88) | 96 (68, 100) | 82 (66, 89) |
| Height (cm) | 161 (154, 167) | 164 (160, 170) | 160 (153, 166) | 166 (163, 166) | 159 (158, 169) |
| BMI (kg/m$^2$) | 31 (28, 36) | 31 (27, 42) | 31 (28, 35) | 36 (24, 36) | 26 (26, 33) |
| Waist circumference (cm) | 101 (96, 112) | 98 (85, 112) | 102 (97, 110) | 118 (96, 124) | 100 (98, 113) |
| Hip circumference (cm) | 106 (100, 117) | NA (NA, NA) | 106 (100, 117) | NA (NA, NA) | NA (NA, NA) |
| Systolic blood pressure (mmHg) | 125 (115, 133) | 119 (109, 133) | 127 (115, 134) | 125 (124, 131) | 134 (114, 189) |
| Diastolic blood pressure (mmHg) | 74 (67, 82) | 77 (67, 83) | 74 (67, 81) | 62 (51, 76) | 80 (70, 86) |

[1] Median (Q1, Q3); n / N (%). NA: not available.

## All of Us

Utilizing the EMR data, which enables precise timeline estimation, we ascertained the chronological order of diagnoses (e.g., diabetes preceding tuberculosis or tuberculosis following diabetes). There were 253 unique individuals who had diabetes diagnosis prior to the tuberculosis diagnosis, 18 unique individuals who had both diagnoses recorded simultaneously, and 213 individuals whose tuberculosis diagnosis was registered before the diabetes diagnosis (Table 2). Thus, there were a total of 484 unique individuals with comorbid diabetes and tuberculosis (Table 3). For AoU, the criteria for diabetes and tuberculosis were based on ICD-10 codes only (Supplementary Table 2).

Table 2
Description of the analytical sample in All of Us stratified by which disease (diabetes or tuberculosis) was recorded first.

| **Variables** | **T2D diagnosed before TB**<br><br>**N = 253**[1] | **TB diagnosed before T2D**<br><br>**N = 213**[1] |
|---|---|---|
| Sex | | |
| *Men* | 99 / 253 (39.1%) | 72 / 213 (33.8%) |
| *Women* | 146 / 253 (57.7%) | 137 / 213 (64.3%) |
| *Missing* | -- | -- |
| Race/Ethnicity | | |
| *Black* | 69 / 253 (27.3%) | 56 / 213 (26.3%) |
| *White* | 53 / 253 (20.9%) | 43 / 213 (20.2%) |
| *Mexican/Other Hispanic* | -- | -- |
| *Other/Multiracial* | 32 / 253 (12.6%) | 27 / 213 (12.7%) |
| *Missing* | 99 / 253 (39.1%) | 87 / 213 (40.8%) |
| Ethnicity | | |
| *Non-Hispanic* | 146 / 253 (57.7%) | 121 / 213 (56.8%) |
| *Hispanic* | 102 / 253 (40.3%) | 86 / 213 (40.4%) |
| *Missing* | -- | -- |
| Education | | |
| *< High School* | 32 / 253 (12.6%) | 26 / 213 (12.2%) |
| *High School Grad* | 67 / 253 (26.5%) | 66 / 213 (31.0%) |
| *Some College/AA* | 115 / 253 (45.5%) | 85 / 213 (39.9%) |
| *College+* | 32 / 253 (12.6%) | 23 / 213 (10.8%) |
| *Refused/DK* | -- | -- |
| Family history of DM | | |
| *Yes* | 42 / 70 (60.0%) | 30 / 57 (52.6%) |
| *Missing* | 28 / 70 (40.0%) | 27 / 57 (47.4%) |
| Fasting Glucose (mg/dl) | 119.09 (37.75) | 113.27 (40.90) |
| HbA1c (%) | 7.52 (2.49) | 6.92 (2.04) |

| Variables | T2D diagnosed before TB<br><br>N = 253[1] | TB diagnosed before T2D<br><br>N = 213[1] |
|---|---|---|

[1] n / N (%); Mean (SD)

Cells with counts < 20 are suppressed and displayed as "--" in accordance with All of Us data dissemination policy. The following groups were fully suppressed: tuberculosis and diabetes diagnosed with the same date (n = 18) because there were < 20 cases.

Table 3
Description of the analytical sample in All of Us.

| **Variables** | **Summary Statistics (All of Us)**[1] |
| --- | --- |
| Sex | |
| *Men* | 181 / 484 (37.4%) |
| *Women* | 291 / 484 (60.1%) |
| *Missing* | -- |
| Race/Ethnicity | |
| *Black* | 130 / 484 (26.9%) |
| *White* | 101 / 484 (20.9%) |
| *Mexican/Other Hispanic* | -- |
| *Other/Multiracial* | 62 / 484 (12.8%) |
| *Missing* | 191 / 484 (39.5%) |
| Ethnicity | |
| *Non-Hispanic* | 280 / 484 (57.9%) |
| *Hispanic* | 192 / 484 (39.7%) |
| *Missing* | -- |
| Education | |
| *< High School* | 59 / 484 (12.2%) |
| *High School Grad* | 136 / 484 (28.1%) |
| *Some College/AA* | 208 / 484 (43.0%) |
| *College+* | 59 / 484 (12.2%) |
| *Refused/DK* | 22 / 484 (4.5%) |
| Family history of DM | |
| *Yes* | 77 / 133 (57.9%) |
| *Missing* | 56 / 133 (42.1%) |
| Fasting Glucose (mg/dl) | 115.66 (39.39) |
| HbA1c (%) | 7.22 (2.27) |

[1]n / N (%); Mean (SD).

| Variables | Summary Statistics (All of Us)[1] |
|---|---|
| [1]Cells with counts < 20 are suppressed and displayed as "—" in accordance with All of Us data dissemination policy. | |

## DISCUSSION

### Main Findings

Utilizing four data resources, including one national health survey (NHANES), two population-based longitudinal cohorts (CARDIA and SOL), and a dataset with EMR (AoU), we developed a unique data infrastructure to simultaneously study diabetes and tuberculosis. By combining SOL, CARDIA, and NHANES, we identified 116 unique individuals who met the criteria for both diabetes and tuberculosis. In AoU we identified 484 unique individuals with comorbid diabetes and tuberculosis. We provide user guides and analytical code for researchers to access pragmatic tools enabling them to effectively and efficiently leverage these data resources to advance their research at the intersection of diabetes and tuberculosis.

### Strengths and Limitations

In a comprehensive search of population-based data resources, historically primarily utilized for advancing cardiometabolic health, we have shown their relevance in studying the diabetes-tuberculosis comorbidity. We provide readers with pragmatic tools, including guidelines and R code, to effectively utilize the four datasets.

However, this work has limitations to acknowledge. First, we focused exclusively on US-based datasets. Other international resources, such as the UK Biobank, may yield an even larger pooled dataset. Following a data pooling study design, future work should seek to bring together more datasets about diabetes and tuberculosis worldwide. Second, despite pooling three large population-based datasets (SOL, CARDIA, and NHANES), the number of observations meeting criteria for both diabetes and tuberculosis was relatively small. Although a limitation, this still provides further value to our work because we delivered a pooled data resource that can, at the very least, be used for exploratory analysis or as preliminary data. Of note, accessing the datasets herein analyzed is free. Third, mostly likely because SOL, CARDIA, and NHANES were not specifically designed to study tuberculosis, we did not have detailed information to assign the organ affected by tuberculosis (i.e., respiratory tuberculosis versus otherwise). Nonetheless, we argue that most cases would be pulmonary tuberculosis as it is the most common organ affected. Fourth, we combined both waves of NHANES, assuming that there are no duplicate individuals, unlike CARDIA and SOL, which are cohort studies following the same individuals. This is a reasonable assumption for NHANES, as it is unlikely that, through a complex random probability sampling across the US, the same person will be selected twice. Fifth, we did not report empirical results, such as associations or further descriptive analytics. Our work focused on delivering a new data resource, including user-friendly, yet detailed guides, along with analytical code for the research community to conduct empirical research. We trust this resource, and this manuscript without empirical results, will be useful for the scientific community and researchers exploring diabetes and tuberculosis, and looking for open-access datasets for this subject. Sixth, in all datasets, self-reported diagnosis or ICD-10 codes (AoU) were used to define tuberculosis. This can result in underreporting, for instance due to recall or social bias (i.e., individuals forgetting their tuberculosis diagnosis or preferring not to disclose it). Therefore, for SOL, CARDIA, and NHANES, it is plausible that we have missed some cases. Conversely, for AoU, we contend that tuberculosis is a severe diagnosis that, in a healthcare system like in the US, would have low probabilities of being undiagnosed, thereby not being recorded in the EMR. Seventh, we cleaned and harmonized a limited set of variables, primarily pertaining to diabetes, tuberculosis, and demographics. The datasets we leveraged encompass a larger number of variables. Interested parties can utilize our tools to obtain the basic variables and then extract the variables required for their needs including long-term fatal and non-fatal outcomes.

## Potential Applications

While we have conducted a comprehensive data cleaning process and implemented a transparent pipeline to deliver a diverse dataset for the study of diabetes and tuberculosis, we acknowledge that the resulting dataset is relatively small. The number of observations with both diabetes and tuberculosis is limited. However, this dataset remains a valuable resource.

At a minimum, this pooled dataset can be utilized for exploratory analysis and to test hypotheses, associations, and determinants. Additionally, this resource can be employed for preliminary analysis and evidence generation for funding applications. Furthermore, teaching and mentoring, particularly in the fields of data science and data pooling, can benefit from this resource.

A notable advantage of our data resource lies in its utilization of two population-based cohorts (SOL and CARDIA) and an EMR resource (AoU). This combination presents extraordinary opportunities. For instance, it enables the study of early determinants of the diabetes-tuberculosis comorbidity. This can be achieved by merging baseline and earlier rounds of the cohort studies. Similarly, it allows for the study of long-term outcomes by leveraging available follow-up rounds and future rounds. SOL and CARDIA are active cohorts with plans to deliver more follow-up rounds. AoU, with the EMR inherent property of analyzing outcomes and events recorded before and after any index condition (e.g., diabetes-tuberculosis), also provides a unique perspective.

Another area of particular interest, and as a specific example, is the study of diabetes phenotypes in relation to tuberculosis. The concept of diabetes phenotypes,[12–14] defined as subgroups of people with diabetes that exhibit distinct responses to certain treatments and progress at varying rates towards complications, has primarily been used to study noncommunicable complications. However, the relationship between these phenotypes and tuberculosis is unknown. For instance, which phenotype carries the highest risk of tuberculosis or any other infectious diseases, and vice versa? Are individuals with a history of tuberculosis more likely to develop diabetes of a specific phenotype through different inflammatory pathways? This evidence can improve disease prevention and diabetes care in the context of infectious diseases.

### Conclusions

We harmonized four data resources (SOL, CARDIA, NHANES, and AoU) to support research on diabetes and tuberculosis, a well-established and significant comorbidity of great interest to researchers in the fields of chronic noncommunicable diseases and infectious diseases. By doing so, we provide the research community with guides and protocols, as well as analytical tools, to efficiently utilize these data resources for studying diabetes and tuberculosis. Additionally, we demonstrated that leveraging available data resources, which are predominantly used for research on noncommunicable diseases, is feasible to expedite the field and integrate both noncommunicable and infectious diseases.

**TABLES**

### Declarations

## DISCLOSURES

**Use of AI**: The authors drafted the initial version of the manuscript. Subsequently, we employed the built-in writing tools available on MacOS (Writing Tools > Professional) to enhance the quality of the manuscript. Finally, the authors verified and revised the AI-refined version. The authors assume full responsibility for the content of the manuscript.

## Data and code sharing

All the resources developed as part of this project are available as supplementary materials. Due to data sharing agreements and the requirement for AoU to complete research ethics courses, we are unable to share the datasets (SOL, CARDIA, AoU). However, our resources provide a comprehensive and step-by-step guide for interested parties to clean and harmonize these datasets. Users will not need to undertake the time-intensive task of identifying variables and cleaning them across datasets because this has been accomplished.

## Conflict of interests:

None.

**Trial**

Not a trial study.

## Funding:

This work was supported by the Emory/Georgia TRAC grant (P30AI168386).

## Acknowledgements:

This manuscript was prepared using SOL and CARDIA Research Materials obtained from the NHLBI Biologic Specimen and Data Repository Information Coordinating Center (BioLINCC) and does not necessarily reflect the opinions or views of SOL, CARDIA or the NHLBI. We gratefully acknowledge All of Us participants for their contributions, without whom this research would not have been possible. We also thank the National Institutes of Health's All of Us Research Program for making available the data examined in this study.
